# GIS-based approaches on the accessibility of referral hospital using network analysis and the spatial distribution model of the spreading case of COVID-19 in Jakarta, Indonesia

**DOI:** 10.1186/s12913-020-05896-x

**Published:** 2020-11-20

**Authors:** Florence Elfriede Sinthauli Silalahi, Fahrul Hidayat, Ratna Sari Dewi, Nugroho Purwono, Nadya Oktaviani

**Affiliations:** Research Division of Geospatial Information Agency of Indonesia, Jalan Raya Bogor Km. 46, Cibinong, Bogor, Jawa Barat 16911 Indonesia

**Keywords:** Service area, Origin-destination cost matrix, Standard deviational ellipse, Spatial distribution, Network analysis, GIS, COVID-19, Referral hospital, Health care

## Abstract

**Background:**

The outbreak of the novel coronavirus (COVID-19) has rapidly spread, causing million confirmed cases, thousands of deaths, and economic losses. The number of cases of COVID-19 in Jakarta is the largest in Indonesia. Furthermore, Jakarta is the capital city of Indonesia which has the densest population in the country. There is need for geospatial analysis to evaluate the demand in contrast to the capacity of Referral Hospitals and to model the spreading case of Covid-19 in order to support and organize an effective health service.

**Methods:**

We used the data from local government publicity for COVID-19 as trusted available sources. By using the verifiable data by observation from the local government, we estimated the spatial pattern of distribution of cases to estimate the growing cases. We performed service area and Origin-Destination (OD) Cost Matrix in support to existing referral hospital, and to create Standard Deviational Ellipse (SDE) model to determine the spatial distribution of COVID-19.

**Results:**

We identified more than 12.4 million people (86.7%) based on distance-based service area, live in the well served area of the referral hospital. A total 2637 positive-infected cases were identified and highly concentrated in West Jakarta (1096 cases). The results of OD cost matrix in a range of 10 km show a total 908 unassigned cases from 24 patient’s centroid which was highly concentrated in West Jakarta.

**Conclusions:**

Our results indicate the needs for additional referral hospitals specializing in the treatment of COVID-19 and spatial illustration map of the growth of COVID-19′ case in support to the implementation of social distancing in Jakarta.

**Supplementary Information:**

The online version contains supplementary material available at 10.1186/s12913-020-05896-x.

## Background

The outbreak of the novel corona virus (COVID-19) has rapidly spread as a global pandemic, and causing million confirmed cases, thousands of deaths, and economic losses. The World Health Organization (WHO) declared the spread of the novel corona virus COVID-19 as a very high global level on risk assessment with total 2,241,359 confirmed cases and 152,551 deaths reported on April 19, 2020. WHO has released operational planning guidelines to support country preparedness and response, guidance on the use of masks in communities, during home care, and in healthcare settings, and further advice for the public [1].

**Fig. 1:**
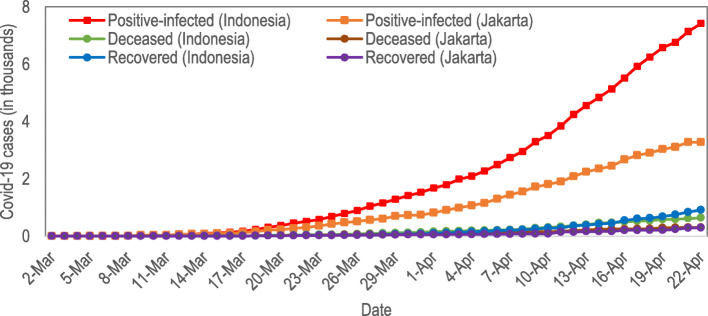
Graph depicting the increasing trend of COVID-19 cases between Jakarta and the national level (source: Provincial Government of DKI Jakarta [3])

The first Indonesia’ case of COVID-19 was identified on March 2, 2020, in Depok, West Java. Later, the number of cases rapidly increased, resulting in 8607 cases in national level including 720 deaths as of April 25, 2020 ([2]). Depok is geographically adjacent to Jakarta, the capital city of Indonesia. The outbreak was identified on March 3, 2020, in Jakarta. Its explosive outbreaks following human mobility patterns. Jakarta has a dense population and has transportation links, including airplanes, trains, interstate buses, and private transportation modes. The number of cases of COVID-19 in Jakarta is the largest in Indonesia, with the total number of confirmed COVID-19 cases is at 3681, including 350 deaths in Jakarta as of Apr 22, 2020 (Fig. 1).

The outbreak of COVID-19 has taken not just lives, but also has devastated workers’ hours and earnings in Indonesia. The economic impact of COVID-19 in Indonesia is fundamentally affecting macro-economic stability and employment. The retail industry in seven cities have been affected by COVID-19, with the five most being impacted are West and Central Jakarta, South Tangerang – Banten Province, Depok and Bandung - West Java Province. The biggest decline with a total 32% fall in daily earnings per outlet was recorded in West Jakarta. According to an International Labour Organization (ILO) report, the outbreak of COVID-19 is expected to wipe out 6.7% of working hours globally in the second quarter of 2020, this is equivalent to 195 million full-time workers [4].

COVID-19 attacks the lungs primarily and transmitted through close contact. The essential needs of health service are being confronted with rapidly increasing demand generated by the COVID-19 cases which grow every day. The Indonesian Ministry of Health has released a national list of 132 referral hospitals, through a decree of the Minister of Health of Indonesia number HK.01.07/MENKES/169/2020 related to the establishment of referral hospitals of certain emerging infection disease, to optimize treatments and health services for infected patients that cover all regencies and cities in Indonesia. For limiting direct mortality and avoiding increased indirect mortality from an outbreak, a prepared and well-organized health service must be provided by Government of Indonesia to maintain an equitable access to essential treatments.

Health systems in several countries are now struggling defeat COVID-19. Some of them approach to collapse. When health systems are overwhelmed due to high demand, both direct mortality and indirect mortality rates increase dramatically. Nowadays, many researchers compete to found some solutions i.e., the tool for estimating the capacity of health system to manage COVID-19 cases per day in the certain catchment area [5]. The accessibility of referral hospitals and their capacities are great importance for protecting the basic human right to health care and maintaining social stability [6, 7], especially related to spreading of infectious diseases that happen at any time, because the consequences are too serious [8]. Government needs to maintain population trust in the health system to provide essential needs and to control infection risk [9–11]. Related to supply of healthcare facility, a study found that a critical bed capacity across Asia varied widely depending on the country income, and Indonesia has 2.7 critical beds per 100,000 population (the 8th lowest ranks of 23 Asian countries) [12]. Then the escalation of COVID-19’s positive cases becoming demand surges that shocking healthcare facilities in this pandemic. Short-term demand forecasting is needed using bunch of data from around the world by considering population density [13].

To strengthen medical response and infection control practices, local or regional health offices should prepare a list of COVID-19 patients in their respective area or review the list of patients through the Public Health Emergency Operating Center (PHEOC). Indonesian Ministry of Health has issued a Decree number HK.01/07/MENKES/446/2020 on reimbursement procedures for hospitals treating COVID-19 cases and this is in line with a Decree of Minister of Health number HK.01.07/MENKES/413/2020 on the guidelines for prevention and control of COVID-19. Hospitals that can claim their services in handling COVID-19 patients are hospitals that provide certain emerging infectious disease services such as referral hospitals and other hospitals that have facilities for COVID-19 patients. The services include service administration, accommodation (rooms and services in the emergency room, inpatient rooms, intensive care rooms, and isolation rooms), doctor services, surgery, the use of ventilators, laboratory diagnostic and radiology according to medical indications, medical consumables, medication, medical devices including personal protective equipment, ambulance, human corpse handling and other health services according to medical indications. So, in order to get full service without worry for the payment, COVID-19 patients must get treated in hospitals that provide certain emerging infectious disease services such as referral hospitals [14, 15].

The health service criteria as per Decree number HK.01/07/MENKES/446/2020 are as follows, 1) criteria for outpatients: a) suspected patients with or without comorbidity should provide evidence of routine blood laboratory test and chest x-ray images. The chest x-ray is excluded for pregnant women and patients with certain medical conditions, including mental disorder and anxiety, as evidenced by a written statement from medical doctor, and b) COVID-19 confirmed patients with or without comorbidity should provide evidence of the results of rapid test-Polymerase Chain Reaction (PCR) laboratory test from hospitals or from other health care facilities; 2) criteria for inpatients: a) suspected patients who are sixty years old with or without comorbidity, patients who are younger than sixty years old with comorbidity, and patients with severe acute respiratory infection (ARI)/severe pneumonia who need hospital care and without other causes based on clinical analysis; b) probable patients, and; c) confirmed patients, includes asymptomatic confirmed patients, who do not have facilities for self-isolation at their residence or public facilities prepared by the Government as evidenced by a written statement from the head of the community health center (*Puskesmas*), confirmed patients without symptoms and with comorbidity, and confirmed patients with mild, moderate, or severe/critical symptoms; and d) suspected/ probable/ confirmed patients with co-incidence. The criteria for outpatients and inpatients apply to Indonesian citizens and foreign citizens, including health workers and workers who have contracted COVID-19 due to work, who are treated in hospitals in the territory of the Republic of Indonesia. Besides, the patient’s identity must be proven by 1) for foreigners: passport, temporary stay permit (KITAS) or UNHCR identification number; 2) For Indonesian citizens: citizenship identification number (NIK), family card, or a written statement from the village; 3) For displaced persons (*orang terlantar*): a written statement from the social service. In the event that the identity documents cannot be provided, proof of identity can be a written statement on patient data signed by the head of local health offices and stamped by the local health offices. The statement is requested by the hospital to the local health offices. In the event that the documents cannot be provided, proof of identity can be a guarantee letter from head of the hospital [15].

Beside of implementation of Decree of Minister of Health number HK.01/07/MENKES/446/2020 and Decree of Minister of Health number HK.01.07/MENKES/413/2020 on the national level, the Governor of the DKI Jakarta has issued regulations to prevent the spreading of COVID-19 by implementing massive restrictions (*PSBB – Pembatasan Sosial Berskala Besar*) in DKI Jakarta by Regulation No. 33 of 2020 on the Provincial level. The regulation of PSBB has implemented started on April 9, 2020. The purposes of this regulation are to restrict certain activities and movements of people and/or goods in suppressing the spread of COVID-19, to increase anticipation of the development of the spread escalation COVID-19, to strengthen health management efforts due to COVID-19, and to deal with the social and economic impacts of the spread COVID-19.

Many literatures have discussed about the significant effect that is being caused by the COVID-19 i.e., the limitations and the needs of improvement on infrastructure and utility [7, 12, 16], participation of community [6, 17], and human movement by tracking and movement modelling [9, 10, 18]. But, during this pandemic, it is important to have information on how the condition of existing health care capacities, especially Referral Hospital for COVID-19 in Jakarta in contrast to the demand. Because, the demand is growing day by day, and there are issues about rejections of COVID-19 patients from some local hospitals in other region of Indonesia. So, in this research, it is critical to conduct site selection, routing, and statistical analysis to know, not only the existing demand and referral hospital capacities, but also to give prediction on the best route from patients to all the possible Referral Hospitals in the certain distances and/or can be reached in the certain time. In addition, it is important to have information on the additional hospital locations recommendation with their capacities, and to explain where the region with the highest prediction growth cases of COVID-19 by statistical model, so the local government and local people strictly implement a social distancing and the health protocol in their region. Most of death cases of COVID-19 was identified accompanied by at least an underlying comorbidity in patients, such as hypertension, diabetes mellitus, respiratory diseases such as asthma and chronic obstructive pulmonary disease [19]. To make cautious analysis about service areas with certain assumptions distance and time travel, in contrast to supply of healthcare facilities at our study area, each type analysis that we performed was carried out by a geospatial approach, particularly using geographical information system (GIS) network analysis and modelling.

A common task in spatial analysis is how to estimate travel time and distance from a set of origins to destinations in a network. We used Origin – Destination (OD) cost Metrix as a feasible solution space and meets demand estimation flow with a definite constraint ([18, 20–23]) that we discuss on method section. Besides, we used SDE model as a long serve technique for analysing concentration tendency of concerned features, orientation, dispersion trends, and distribution differences, delineating the geographic distribution of concerned features, and providing a comparable estimate of the individuals’ activity spaces ([24–27]). Our paper contained the report of the growth cases of COVID-19 in Jakarta, Indonesia and our objectives are as follow: 1) to introduce geographic distribution of COVID-19 based on GIS spatial technology; 2) to recommend the nearest healthcare facilities by service area and OD Cost Matrix; and 3) to create SDE model of the study area.

## Methods

### Study area

**Fig. 2:**
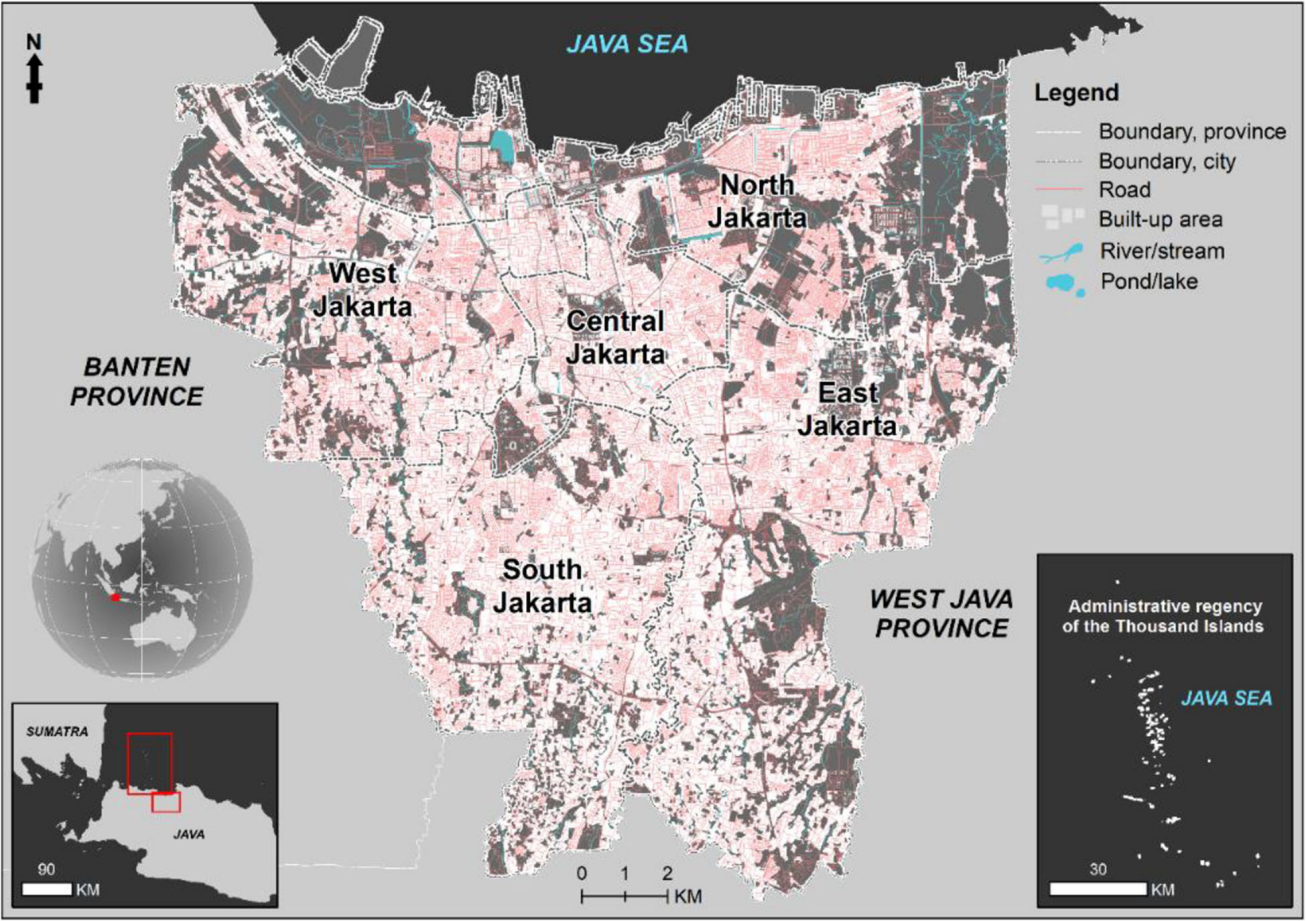
The 2014 topographic map of DKI Jakarta published at scale 1:25,000 obtained from the Indonesian Geospatial Information Agency

Jakarta is the capital city of Indonesia located in Java Island which has the densest population in the country. The central point of the area is at geographical coordinates 6°12′29″ S and 106°50′20″ E. Jakarta as the state capital has a special status and special autonomy granted under Law of Indonesia Number 29 Year 2007. Administratively, the area of Jakarta is divided into five municipalities and one district administration [28]). The administrative regions below are divided into 44 sub districts and 267 administrative villages (Fig. 2). Its economic structure in 2018 was dominated by wholesale and retail trades, repair of motor vehicles, and motorcycles sectors, that reached 16.93% of the total Gross Domestic Regional Bruto of the DKI Jakarta Province. Total workers in Jakarta was 25,121 people in 2017, not included the number of job seekers, who come from, for instance, Depok, Bogor, Tangerang, and Bekasi, which are regions adjacent to Jakarta. Its population density in 2017 was 15,663 people/km^2^ with the population growth rate of 0.94% per year. In this case, West Jakarta has the highest population density in the amount of 19,516 people/km^2^ [28]).

### Data collection

The study began with the compilation of feature datasets such as road lines, administrative boundaries, hospital and settlement layers from topographic map of Jakarta at scale of 1:25,000 and the 2015 population density dataset with a spatial resolution of 100 × 100 m^2^ from the Indonesian National Board for Disaster Management [29]. All the data are public domain provided by Government of Indonesia. A total of 261 centroid of villages representing the position of COVID-19 patients observed were mapped in the area as of March 22, 2020. The local government used the centroid of the administrative regency to show series of confirmed cases of COVID-19. The centroid or the geometric centre, is a point feature representing a vector data (multipoint, line, and area features). The centroid is located inside or contained by the bounds of the input feature. Since Jakarta is an urban big city, the density of geometric centre will be distributed fairly since the open area is limited. We used centroids of villages as a patient origin based on positive-infected cases report on each village. In Indonesia, we have a restricted protocol of health data publication and the access to data is limited. So, in order to make this research represents actual condition, we used centroid of villages in Jakarta as the origin with an update attribute data on each centroid as per April, 16, 2020 In this research, majority of our maps are obtained from data analysis using Network Analyst tool extension in ArcGIS 10.5 and Spatial Statistics toolbox in ArcGIS Pro 2.5. We used ArcGIS Desktop Advanced 10.5 concurrent use and Arc GIS Pro Advanced under subscription ID 2422920474 owned licensed by Geospatial Information Agency of Indonesia.

We obtained the daily series of confirmed cases of COVID-19 in Jakarta from the first case identified on March 3, 2020, to April 16, 2020, that are publicly available from Provincial Government of DKI Jakarta [3]). The total number of confirmed cases as of April 22, 2020, are 3681 positive-infected cases, with 1947 patients was hospitalized, 334 patients was discharge, 1050 cases with self-quarantine status, and 350 patients died. In our research, we focussed only on analysing the positive-infected cases. The total number of confirmed and positive-infected cases as of April 16, 2020, as well as classification cases based on gender and age were presented in Table 1.

**Table 1:** The total number of positive-infected cases as of April 16, 2020, as well as the distribution by gender and age [3])

Classification		Female	Male
Age	> 60 yrs	254 (9.63%)	347 (13.16%)
	50–59 yrs	241 (9.14%)	347 (13.16%)
	40–49 yrs	170 (6.45%)	240 (9.10%)
	30–39 yrs	150 (5.69%)	229 (8.68%)
	20–29 yrs	151 (5.73%)	135 (5.12%)
	6–19 yrs	47 (1.78%)	27 (1.02%)
	<= 5 yrs	4 (0.15%)	7 (0.27%)
	Couldn’t be identified	95 (3.60%)	193 (7.32%)
Total	**2637 (100%)**	**1112 (42.17%)**	**1525 (57.83%)**

**Fig. 3:**
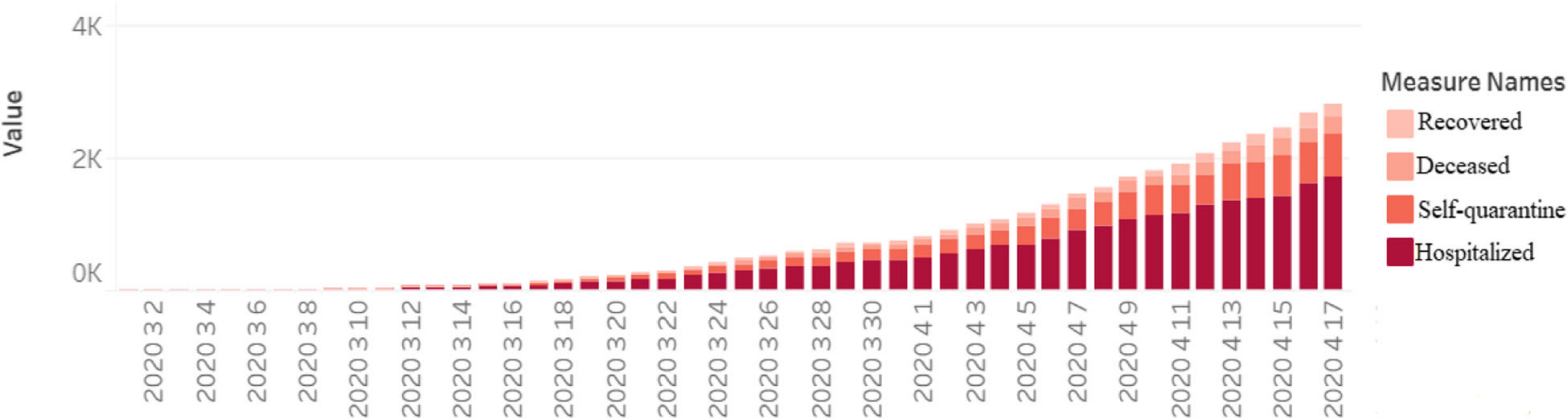
Graph depicting data accumulation for overall COVID-19 cases in Jakarta based on number of recovered, deceased, self-quarantine, and hospitalized report. (source: [3])

The number of COVID-19 cases in Jakarta quickly ascended following an exponential growth trend. We obtained the graph which visualizing data accumulation for the overall COVID-19 cases in Jakarta from March 1, 2020, to April 17, 2020 (Fig. 3).

**Fig. 4:**
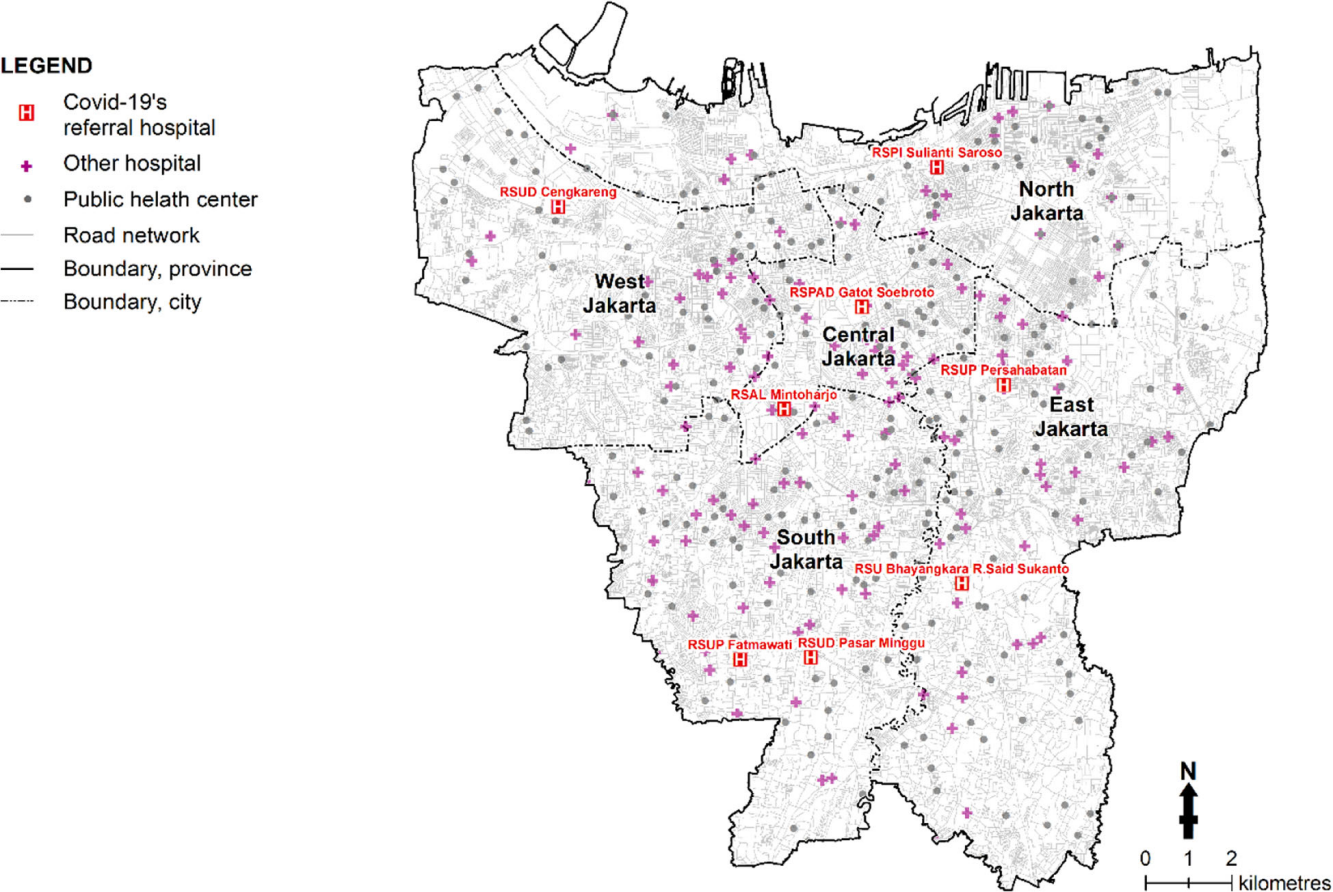
Locations of referral hospital of COVID-19 and other hospitals and community health centres in DKI Jakarta (source: Indonesian Ministry of Health [30])

**Fig. 5:**
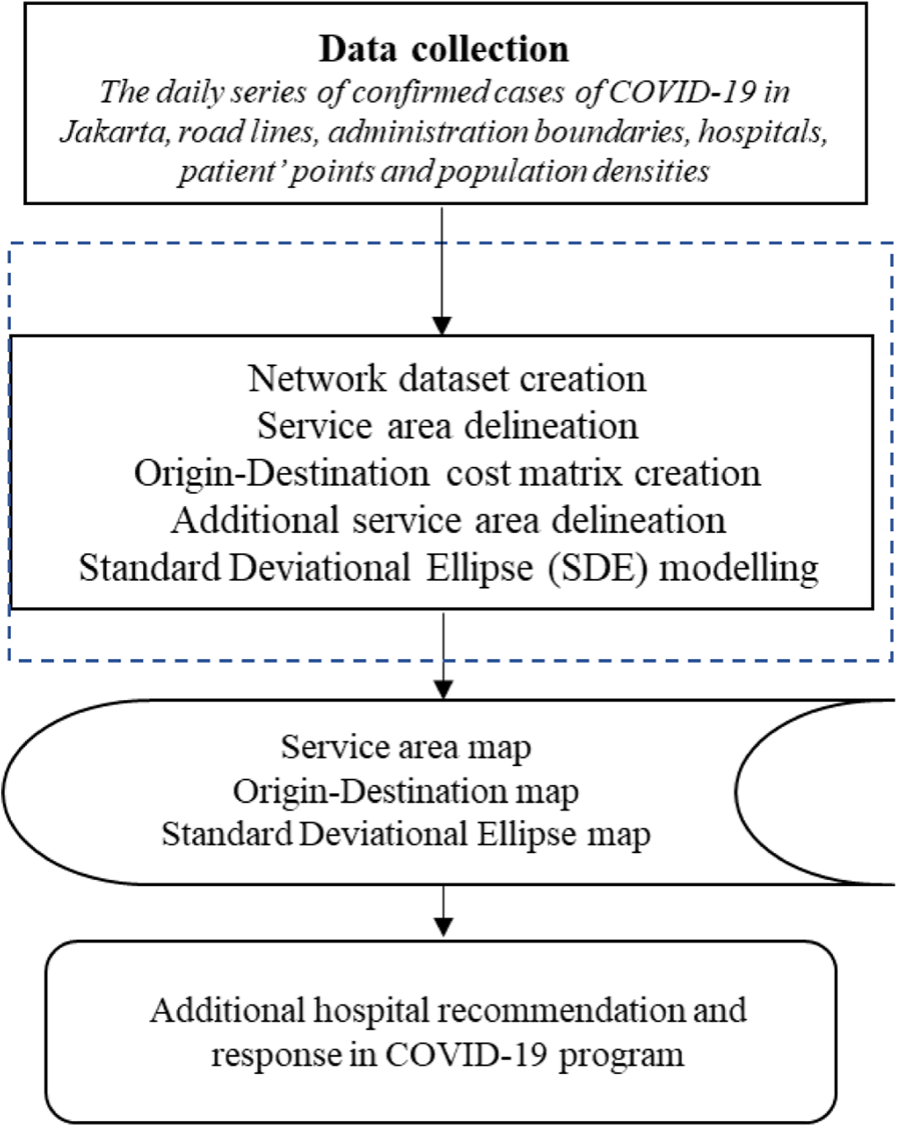
Flowchart of this research process

Besides, we collected referral health care facilities information as data attributes containing related information about infrastructures such as bed allocation for isolation room, bed allocation for Emergency Department, capacity of Intensive Care Unit (ICU), Paediatric intensive care unit (PICU), Neonatal Intensive Care Unit (NICU), Intensive Coronary Care Unit (ICCU), and High Care Unit (HCU) facilities. A total of eight referral hospitals were located in Jakarta (Fig. 4) with details capacity as attached on Additional file 1. We also collected information related to health workers involved in COVID-19 control programs, such as specialists and doctors, as well as other health workers that have been working to assist in the COVID-19 response [31]. Figure 5 represents a general framework of this research including data collection, spatial analysis, the output and recommendation for COVID-19 cases in Jakarta.

Descriptions of each of step are as follow:

1. Network dataset creation

Networks are a specific type of vector data which is primarily composed of edges, junctions, and nodes. Network datasets are used to choose path and commonly used to model transportation networks. Transportation networks are used for modelling interaction such as allocating demand to facilities and modelling travel to facilities [22, 32, 33]). Many methods have been used to model the spatial accessibility to facilities including allocating demand to those facilities such as by using ArcGIS Network Analyst [34, 35], Frequency Ratio and Analytical Hierarchy Process [36], two-step floating catchment model [37, 38], and Space Syntax and location-based method [39]. Network analysis derived from route network data, and delineation of population catchment areas and Referral Hospital, using Geographic information systems (GIS) by travel-time-based and/or distance-based is commonly used and effectively utilized to carry out spatial accessibility of the healthcare services [34, 36–38]. A study related to relationship identification of active mobility activities such as walking, cycling and active transport journeys, with the environmental factors and the pattern of social interaction by spatial models, shows the intersection density and land use are strongly associated with active and public transport accessibility. It also improved by the infrastructure to support active travel and regulation to limit vehicular speed [36].

For this research, we performed an analysis on our road network dataset from the topographical map of Jakarta provided by Geospatial Information Agency, and using the ArcGIS Network Analyst extension. We also created two scenarios in service area delineation and we used not only a finite-size buffer ring around each centroid but we used OD cost matrix for modelling the spatial distribution of travel demand (next step). This road feature dataset has limitation in providing data requirements of network elements to a transportation network such as one-way streets, link impedances, turn impedances, underpasses, and overpasses. Example for CurbApproach, we did not give a specified side, so we permitted it from either side of the vehicle from the origin to be on when the vehicle departs Therefore, we had to make assumptions when performing our service area analysis.

2. Service area delineation

After a road network dataset of road line was built, we generated a service area based on two scenarios to show locations which can be reached within a certain distance or time. The results when using different approaches of the distance or time will influence the optimal service areas to be developed. In fact, both distance-based and time-based model to create the service area is interrelated in defining the quality of health-services in certain locations [40, 41]. By using distance-based scenario, we would like to evaluate healthcare services and knowing the impact of distance to emergency condition of patients. Since the increase of distance might affect less opportunity to survive from certain illness in emergency situation [42]. However, by using only distance in developing the service area tends to produce less optimal results due to possible traffic jams or local traffic pattern [41].

We defined referral hospital locations as facilities or points on a map as the starting point (point of origin) where service areas would be developed. This analysis helps us to evaluate coverage and accessibility based on how much of something is within referral hospitals neighbourhood or region. Service areas are important for evaluating healthcare services, accessibilities, and population-based health indicators for disease burden [43]. The method that we used for service area analysis is network-based [44].

We set two scenarios to develop the service areas, namely: 1) distance-based service area of 5 and 10 km or distance scenario; and 2) time-based service area or driving scenario by using time parameters derived from distance (geometry) and speed data adopted from the Indonesian Ministry of Transportation regulation [45, 46]. Since the most of death cases of COVID-19 was identified accompanied by at least an underlying comorbidity in patients which required quick health treatment and response, we assume the best distance-based service area of 5 and 10 km and based on driving time of 5, 10 and 15 min.

Some justifications were made due to different standard between road classes in the regulation and road classes provided by topographic map. Table 2 shows the speed details for various classes of road in Indonesia based on the Indonesian Ministry of Transportation regulation, while Table 3 presents justifications of the speed of each road class that we made to this research. Here, we modified the speed by including the effect of traffic on the driving speed in Jakarta due to severe congestion in Jakarta. We adopted research conducted by the Directorate General of Land Transportation [46] and set driving speed to 20 km/h for arterial, collector, local and other roads. However, we assumed that there is no congestion in the highway.

**Table 2:** Driving speed detail information for various classes of road in Indonesia that was used to develop service area of each referral hospital [45]

Road classes	Speed (km/h)	Road classes	Speed (km/h)
Highway (maximum)	100	Primary collector road	80
Highway (minimum)	60	Secondary collector road	50
Primary arterial road	80	Primary local road	30
Secondary arterial road	50	Secondary local road	25

**Table 3:** Justification of driving speed chosen for each of road classes provided in the topographic map

Road classes	Speed (km/h)	Speed (km/h) with traffic effect	Notes
Highway	80	80	Average speed of highway
Arterial road	65	20	Average speed of primary and secondary arterial road
Collector road	65	20	Average speed of primary and secondary collector road
Local and other roads	27.5	20	Average speed of primary and secondary local road;
Footpath/Pedestrian way/alley	0	0	As barriers

3. Origin-destination cost matrix creation

Next, we performed an origin-destination (OD) cost matrix analysis to create a matrix showing distances using a network dataset and to assign where confirmed COVID-19′ patients locations to the nearest referral hospital. In this study, Origin-Destination (OD) cost matrix was chosen because it has been used to find a solution closed to real matrix from feasible solution space which meets demand estimation flow with a definite constraint example for traffic planning, management and control [18, 20–23]. The OD cost matrix is a traditional and widely used approach for modelling the spatial and temporal distribution of travel demand [18, 23] aiming to find a solution which meets traffic flow constraints and closed to the real matrix from feasible solution spaces [21, 22]. We performed OD cost matrix analysis using network analyst extension on the Arc Pro 2.5 with a logistic service hosted by ArcGIS Online. By using this logistics, we got a high-quality solver references using worldwide network dataset which is stored in the ArcGIS Online cloud [47].

After the network dataset was set unto the project, we defined settings and constraints as parts of network parameters [33] such as, 1) mode, which refers to the type of transportation and distance versus time. In this, we used rural driving distance; 2) cut-off, which refers to a maximum time or distance within the analysis of target to search for destination. On this research, we set cut-off in a range of 10 km from the origin target to search for destination without barriers; 3) origins, we used 261 centroids of villages as a patient origin based on positive-infected cases report on each village with an update attribute data on each centroid as per April, 16, 2020; 4) destinations, which refers to the number of destinations to find should be set, we did not set a number of destinations, so the output will be the travel times for the paths from each origin to each possible destination; 5) search tolerance, we used 5 m as the input to locate the features on the network, so features that are outside the search tolerance are left unlocated; 6) CurbApproach, we permitted it from either side of the vehicle from the origin to be on when the vehicle departs; and 7) the output, we chose straight line for representing the result paths along the network. The OD cost matrix provides solver output represented as the straight lines on the map, but the values stored in the lines attribute table reflect the network distance from origins to destinations. OD cost matrix output is more appropriate than straight-line cost and it often become input for other spatial analyses [48].

The matrix output is a table which lists the total impedance (could be a distance, time, or money) for the shortest path along the network between each centroid of COVID-19 patients (origins) and each referral hospitals as a demand points (destinations). The distance that is stored in the origin-destination cost matrix is calculated over the street network. Each demand point is assigned to the facility its closest to.

4. Additional service area delineation

Additional service area was developed to find the most accessible hospitals which can be reached by unassigned patients or patients who live in rural area. In this case, we developed 5, 10, 15 min time-based service area from unassigned patients (as origin point). Next, we identified a more accessible near-patient hospital located within these 5, 10, 15 min distance. We put priority to the hospital located within 5 min as we assumed that less easily accessible hospital will increase cost to patients [49], however, the capacity of the hospitals was also part of our consideration.

5. Standard Deviational Ellipse (SDE) modelling

We used the SDE model to investigate spatial distribution in particular of dispersion pattern and direction changes of COVID-19 cases in Jakarta. We employed SDE model tool in ArcGIS to gain a better understanding of the geographical aspects of the COVID-19 phenomena and identify the cause of this event based on its specific geographic patterns in particularly for elliptic spatial scan statistic [24]. In this research, the illustration of the SDE model for certain area was depicted by gradient colour or tone level of the ellipses. A brighter colour indicates a recent data. The characteristic pattern of events illustrates the central tendency of the events to certain direction, in which the axis of SDE will be appeared skewed towards certain direction which indicated its distribution. The SDE model depicting a standard deviation graphs (ellipse) on the *X* and *Y*-axes of features locations which are focussed on the geometric mean value or mean centre for the features. The SDE formula was first suggested by Lefever [50], further was corrected in subsequent publications [51], so that until now it has generally been stated with the following equation:

1 $$ {SDE}_X=\sqrt{\frac{\sum_{i=1}^n{\left({\mathcal{x}}_{\mathcal{i}}-\overline{X}\right)}^2}{n}},{SDE}_Y=\sqrt{\frac{\sum_{i=1}^n{\left({y}_{\mathcal{i}}-\overline{Y}\right)}^2}{n}} $$

where ${\mathcal{x}}_{\mathcal{i}}$ and ${y}_{\mathcal{i}}$ are the coordinates for feature $\mathcal{i}$, { $\overline{X}$, $\overline{Y}$ } represents the mean centre for the features along the Cartesian coordinate system, and *n* is equal to the total number of features. The mean centre is essential to understand average location of many point distributions [27, 50–53]. For measuring the dispersion distribution of original observations, the major axis and minor axis of the SDE can be determined as follows:

2 $$ {\sigma}_x=\sqrt{2\ }\sqrt{\frac{\sum \limits_{i=1}^n{\left(\tilde{x}_{i}\cos \alpha -\tilde{y}_{i}\sin \alpha \right)}^2}{n}},{\sigma}_y=\sqrt{2\ }\sqrt{\frac{\sum \limits_{i=1}^n{\left(\tilde{x}_{i}\mathit{\sin}\alpha -\tilde{y}_{i}\mathit{\cos}\alpha \right)}^2}{n}} $$

**Fig. 6:**
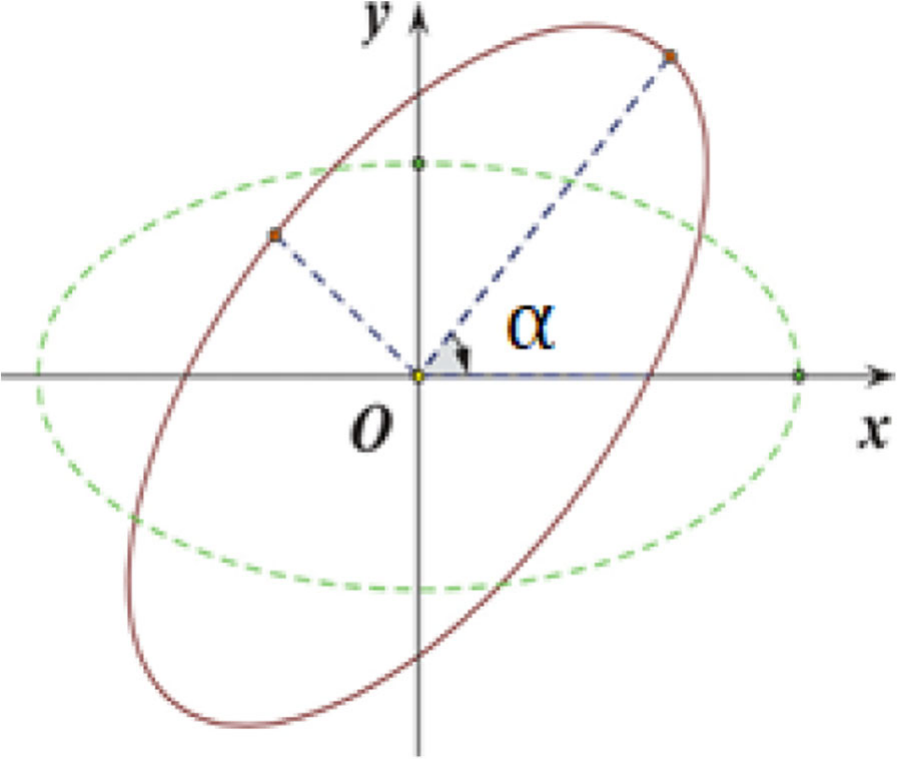
An ellipse which rotates in the direction of the clockwise angle (modified from Wang et al., [27])

Furthermore, the use of weighted data involves an equation of shape to evaluate the ellipse direction [52, 53]. For the angle of rotation, it is calculated as follow:

3 $$ \tan \alpha =\frac{p+q}{r} $$

in which,

4 $$ p=\left(\sum \limits_{i=1}^n\tilde{x}_{i}^2-\sum \limits_{i=1}^n\tilde{y}_{i}^2\ \right) $$

5 $$ q=\sqrt{{\left(\sum \limits_{i=1}^n\tilde{x}_{i}^2-\sum \limits_{i=1}^n\tilde{y}_{i}^2\ \right)}^2}+4\ {\left(\sum \limits_{i=1}^n\tilde{x}_{i}\tilde{y}_{i}\ \right)}^2 $$

6 $$ r=2\sum \limits_{i=1}^n\tilde{x}_{i}\tilde{y}_{i} $$

where $\tilde{x}_{i}$ and $\tilde{y}_{i}$ are the deviations of the *xy*-coordinates from the mean centre (see Fig. 6).

For depicting the dispersion of COVID-19 cases in a range of 23-days over five regions of DKI Jakarta, SDE helps us to obtain descriptive statistics of univariate features around its centre. Using an ArcGIS extension, supposing the features concerned usually follow a spatially normal distribution. However, it is mainly determined by three measures i.e., average location, dispersion (or concentration) and orientation [27]).

## Results

### Service area of referral hospitals

Identification of the movement and transport of people, disease, or goods, its migration patterns and commuting behaviour, is a spatial challenging in visualisation problem. Physical time and space are two dimensions which are considered to define a function of moving objects in transportation. We used GIS to process our map and model. It has not only a strong association for creating a transportation model, but also provided capable environments on management, analysis, and visualization of spatial data, i.e., for an integration of various data sources [32].

**Fig. 7:**
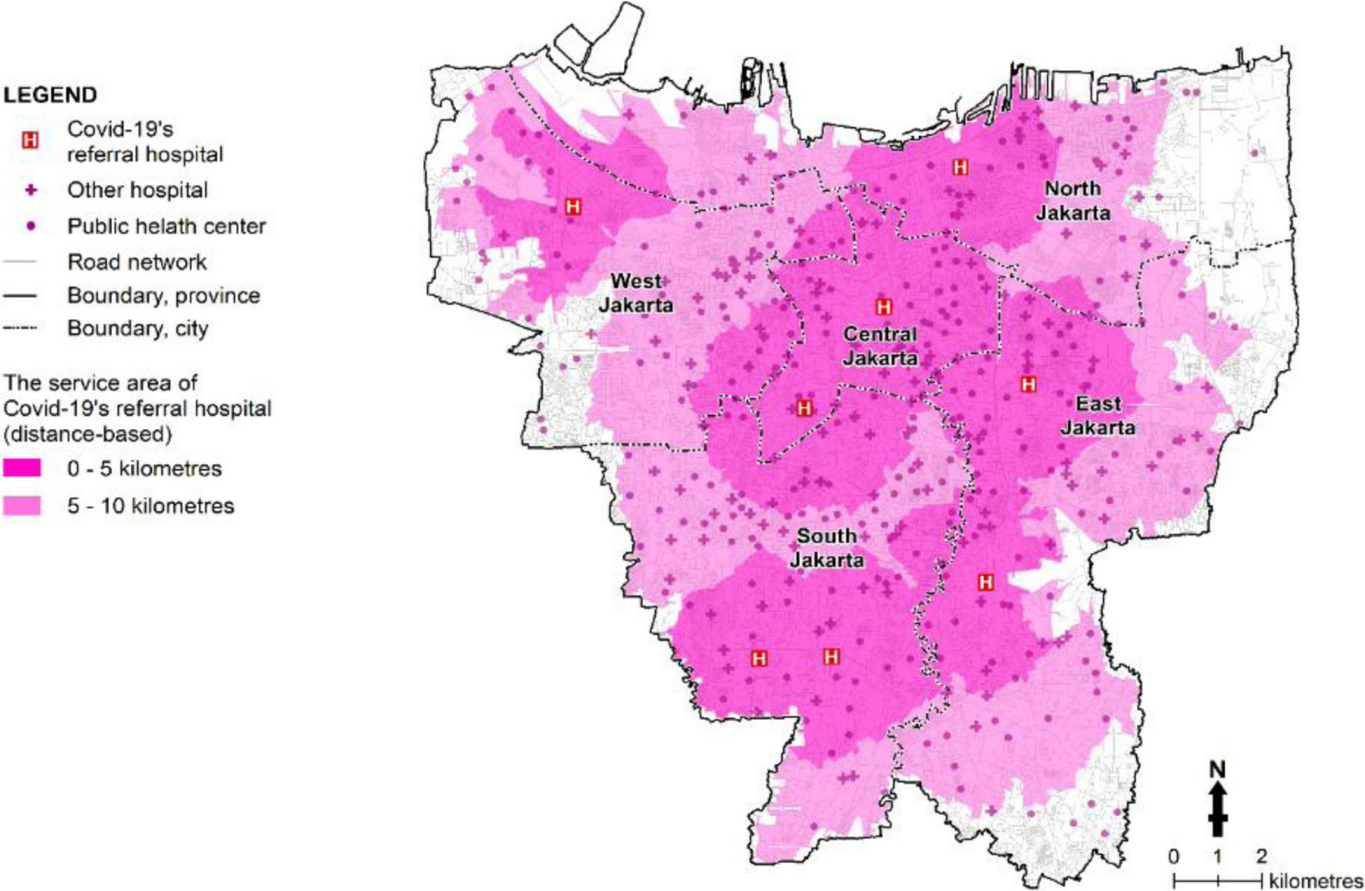
Graph depicting service area of referral hospital of COVID-19 in Jakarta based on distance of 5 and 10 km (source: own elaboration, 2020)

Figure 7 shows the overall distance-based service areas developed for eight referral hospitals. When using 5 km scenario (in dark purple), we can see that all referral hospitals can only cover half of Jakarta Province. Hence, we extended the coverage of the hospitals to 10 km (in light purple). On the one hand, the new coverage covered more people, but on the other hand, it resulted in overlapping service areas due to relatively close position of the referral hospitals. The other main drawback to this scenario was time needed for patients to reach the referral hospitals was remain unknown. Furthermore, previous studies have indicated that it is important to include the actual traffic conditions to the time element such as speed limits, traffic jams, and one-way street [54] in particular for emergency services for COVID-19 patients, for example, patients experiencing shortness of breath.

**Fig. 8:**
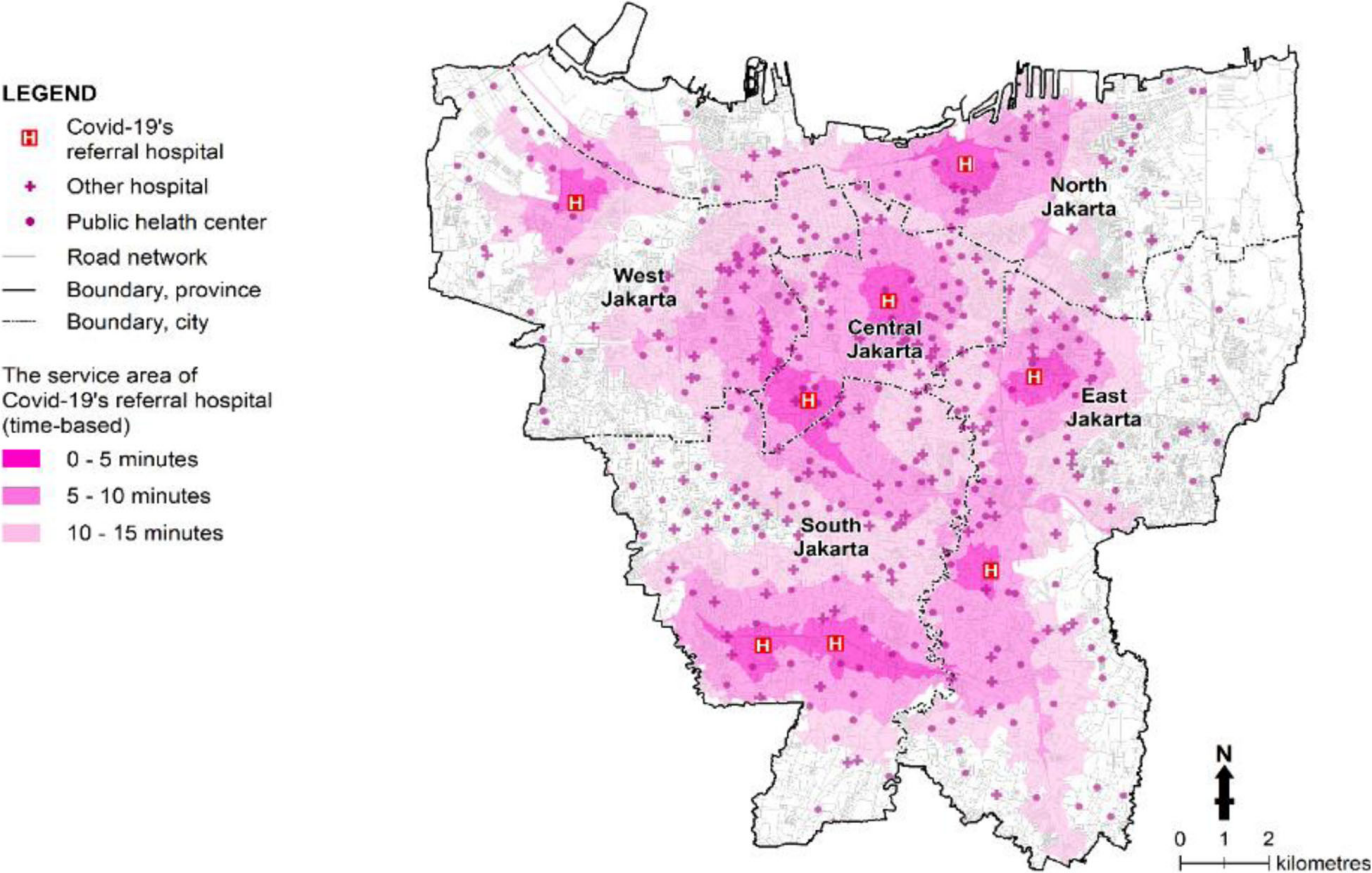
Graph depicting service area of referral hospital of COVID-19 in Jakarta based on driving time of 5, 10 and 15 min (source: own elaboration, 2020)

To overcome such drawback, Fig. 8 presents the second scenario incorporating time components to develop the service area of the referral hospitals. For the second scenario, the calculated service areas of each referral hospital were divided in to three categories: 0–5, 5–10, and 10–15 min. From Fig. 8, we can see that all Central Jakarta and most of South Jakarta were located at the well served area to the referral hospitals. In contrast, there were 55 villages which were not covered by the referral hospitals, representing 21% of the total number of villages in Jakarta. These villages were located in the North, East and West Jakarta. This implies that the local government needs to suggest more referral hospitals to cover people who live far away from the existing referral hospitals.

To further evaluate the effectiveness of both distance-based and time-based scenarios chosen, we superimposed the service areas with the population data so we can obtain the number of people living in certain service areas. With total population of approximately 14.3 million people live in Jakarta, more than 12.4 million people (86.7%) based on distance-based service area, live in the well served area of the referral hospital. Meanwhile, when using the time-based service area, the total number of people who live in the catchment are less than 9.4 million (65.8%), as can be seen in Table 4.

**Table 4:** Number of people living in each service area of the referral hospital

	Scenario	Number of people	Percentage (%) of total population
1	Distance-based	12,401,618	86.7
2	Time-based	9,422,606	65.8

Information in Table 4 only presents a general information regarding number of people who are served by the referral hospitals. We need to evaluate the capacity of existing referral hospital with respect to the number of positive-infected COVID-19 patients. To do so, we superimposed the service areas with the positive-infected cases of COVID-19 as can be seen in Table 5. The term “unserved area” in Table 5 is defined as the area outside the service area of the referral hospitals. Using both scenarios in Table 5, we can see that the area which were best served by the hospital were mainly located in the Central and South Jakarta.

**Table 5:** Number of positive-infected COVID-19 cases in the served and unserved area of the referral hospitals

District	Number of positive-infected COVID-19 cases			
	Distance-based		Time-based	
	Served	Un-served	Served	Un-served
West Jakarta	1054	42	1024	76
Central Jakarta	238	0	229	0
South Jakarta	714	0	661	61
East Jakarta	374	40	263	148
North Jakarta	151	23	119	55

### OD matrix analysis results

**Fig. 9:**
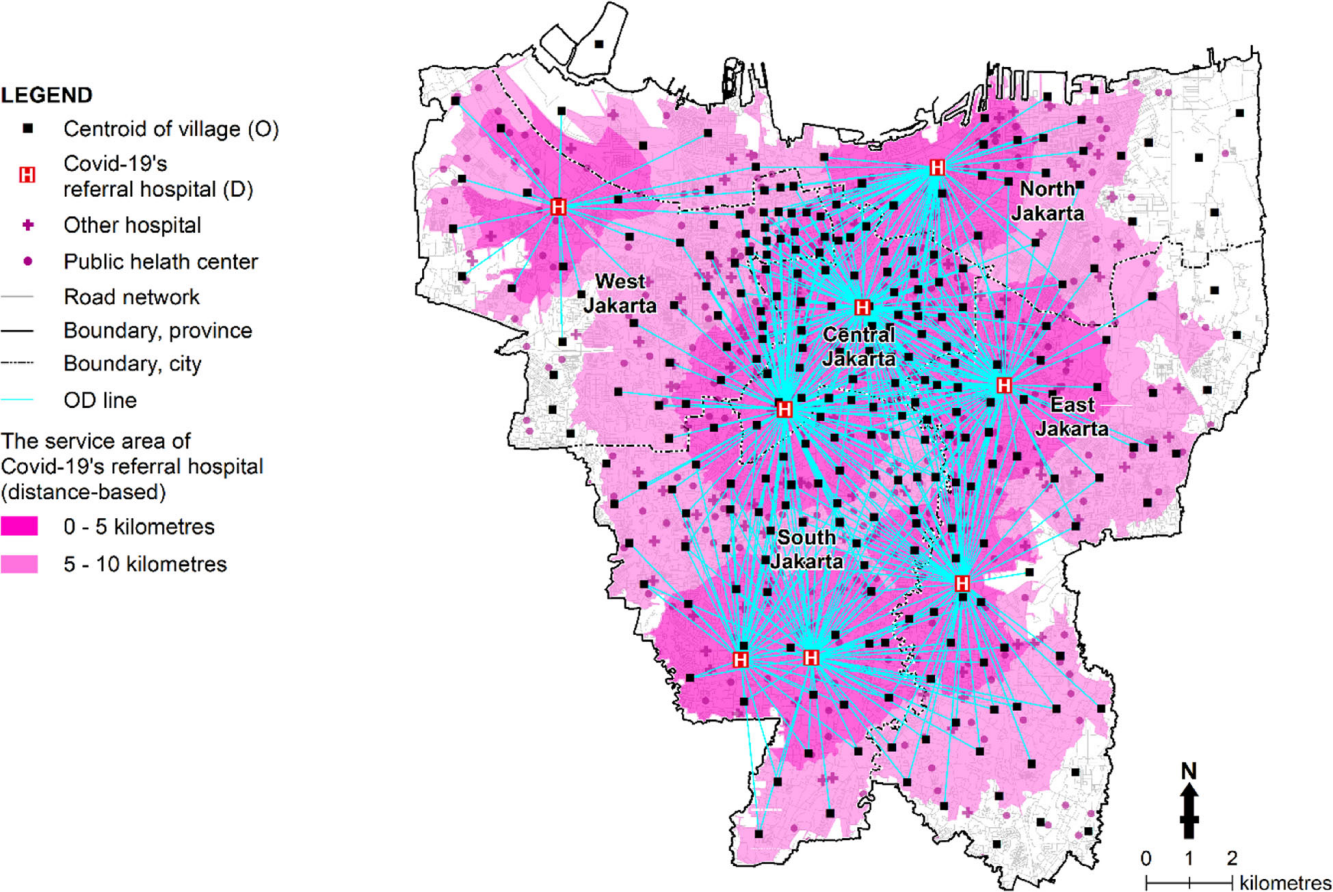
Graph depicting (OD) cost matrix of COVID-19′ positive-infected patient centroids (origins) to the referral hospitals (destinations) in Jakarta in 10 Kilometres (source: own elaboration, 2020)

To evaluate the accessibility of patients to the referral hospitals, we used the OD matrix method as can be seen in Fig. 9. The table of OD cost matrix with cut-off of 10 Kilometres has 549 rows with information on paths to each of the eight referral hospitals. The matrix shows the total travel time, shortest to longest distance, and destination rank, from each origin to destination. Based on this matrix, we derived detailed information regarding the required services that should be given by each referral hospital as in Table 6.

**Table 6:** Total cases of COVID-19 assigned to referral hospitals based on the OD Matrix analysis

ID	Hospital	District	Villages Served	Premiere case	Secondary case	Third case	Fourth case	Fifth case	Total cases
60	RSPI Sulianti Saroso	North Jakarta	60	125	120	62	10	0	317
61	RSUP Persahabatan	East Jakarta	76	177	96	133	12	0	418
66	RSU Bhayangkara Tk. I R. Said Sukanto	East Jakarta	66	238	90	68	26	0	422
62	RSUP Fatmawati	South Jakarta	42	462	130	10	4	0	606
65	RSUD Pasar Minggu	South Jakarta	63	63	572	112	5	0	756
63	RSPAD Gatot Soebroto	Central Jakarta	107	264	201	79	12	4	556
67	RSAL Mintoharjo	Central Jakarta	111	319	169	77	37	0	602
64	RSUD Cengkareng	West Jakarta	21	10	53	9	28	0	100

Note: Premiere, secondary, third, fourth, and fifth cases define as cases from the first, second, third, fourth, and fifth destination rank in the OD Matrix results

From Table 6, we can see that two hospitals in Central Jakarta had been assigned to receive positive-infected COVID-19 patients from various villages within the range of the OD cost matrix range. The hospitals were RSPAD Gatot Subroto and RSAL Mintoharjo which served 264 and 319 premiere cases of COVID-19 from 107 and 111 villages, respectively. Meanwhile, RSUP Fatmawati in South Jakarta had been assigned to serve the highest premiere cases (462 patients) from 42 villages. In contrast, in terms of total positive-infected cases, RSUD Pasar Minggu had been assigned to serve the highest number of positive-infected patients, especially the third cases patients from 63 villages.

**Fig. 10:**
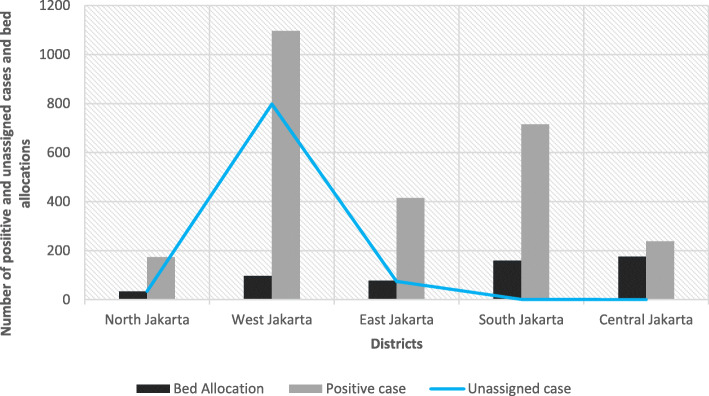
Graph depicting demand in contrast to the capacity of Referral Hospitals and unassigned positive-infected cases generated from OD Cost Matrix in Jakarta in a radius of 10 Kilometres from patients’ centroid

From the results, we also got information that a total 2637 positive-infected cases were concentrated in West Jakarta (1096 cases), following by South Jakarta (715 cases), East Jakarta (414 cases), Central Jakarta (238 cases) and North Jakarta (174 cases) as per April 16, 2020. Besides, there was a total 908 unassigned cases from 24 patient’s centroid which was highly concentrated in West Jakarta in a range of 10 km (see Table 7). We found the highest cases at Village of Jelambar Baru, West Jakarta, with a total 774 positive-infected cases. This centroid of patients had not been assigned to any number of destination (referral hospital). From a total 1096 positive-infected cases in West Jakarta, 798 cases or 72.81% are unassigned. Besides, only one referral hospital in this area (RSUD Cengkareng) and this hospital could not overcome the demand (see Fig. 10). For the second highest cases, we had a total 385 of cases from origin 11, Bintaro, South Jakarta, with OD matrix in a range of 10 km, this centroid of patients had been assigned to destination number 62 (RSUP Fatmawati) and 65 (RSUD Pasar Minggu). Meanwhile, we found one case at Petukangan Utara, South Jakarta which out of OD cost matrix range. Besides the capacity both of RSUP and RSUD Pasar Minggu could not overcome the demand.

**Table 7:** Number of positive-infected COVID-19 versus bed allocations and unassigned cases in each district

Area	Number of villages	Number of positive-infected patients	Bed allocations	Unassigned cases
North Jakarta	31	174	34	34
East Jakarta	65	414	77	75
South Jakarta	65	715	159	1
Central Jakarta	44	238	175	0
West Jakarta	56	1096	97	798

### Additional hospitals

The outbreak of the COVID-19 makes many countries, including Indonesia, struggling with insufficient accessibility, a long waiting times at emergency departments and primary healthcare centres. Increasing in attendance level at emergency departments with acute admissions, meanwhile we are confronting COVID-19, gives adversely impacts and expose patients unnecessarily to the risks of hospital admission. Crowd level at an Emergency department can create risks for patients by causing delays in transport and treatment affecting the patient mortality rate [6, 16]). So, by mapping the capacity of referral hospitals and additional hospitals in contrast with cases distribution, we want to summarize the importance of applying a geographical or spatial perspective in support to primary healthcare centres research and practice. Based on what we have explained above, this would lead to the most equitable and accessible ways possible in, by, and for communities to stand the outbreak (Crooks and Andrews, 2009).

**Fig. 11:**
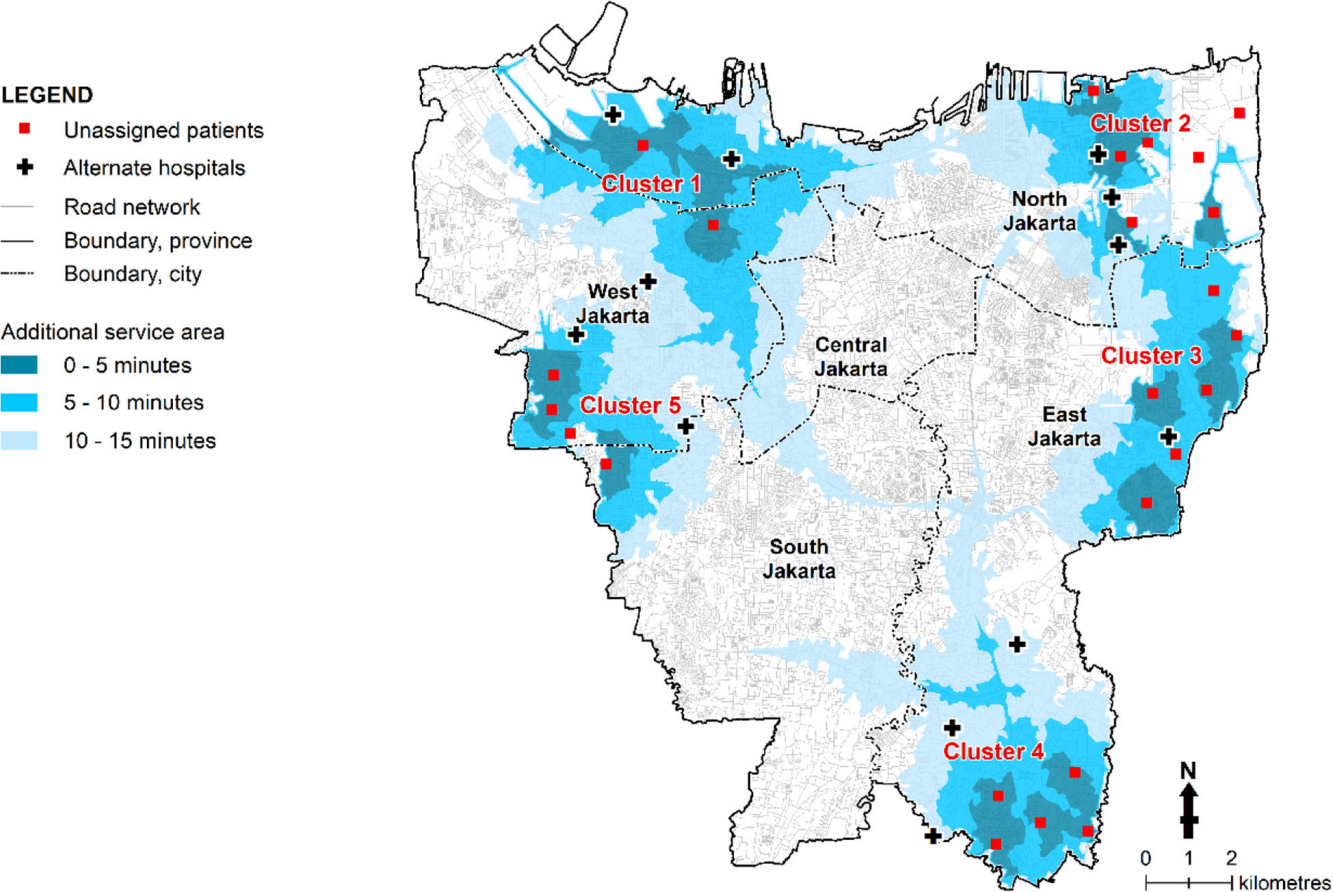
Additional service area developed for unassigned positive-infected patients of COVID-19 using time-based service area with 5, 10, and 15 min travel time from the centroid of patients as the origin (source: own elaboration, 2020)

Figure 11 shows the additional service areas developed from 24 unassigned patient’s centroids. From the results, we can see that in general, there were five clusters for the additional hospitals. Here, we grouped hospitals in clusters since the service area was developed by considering travel time, so it was possible patients were assigned to hospitals in the neighbouring area.

In order to choose which hospitals could be suggested as additional hospitals for the unassigned COVID-19 patients, we superimposed additional hospital data located within 5, 10, and 15 min coverage of the additional centroids. First priority was given to the hospitals located within 5 min-distances from the centroid of unassigned positive-infected patients and we evaluated the capacity of those hospitals as in Table 8. However, if the required hospitals were not available or the capacity of the hospital did not fulfil the required capacity, then we extended our observation to the second layer, 10 min-distances from the origin and so on.

**Table 8:** The number of bed allocations of each alternate hospital in contrast to unassigned patients

Cluster	Name	District	Existing Bed Allocation	Unassigned patient
Cluster 1	RS Pantai Indah Kapuk	North Jakarta	9	783
	RS Akademik Atma Jaya	North Jakarta	16	
Cluster 2	RS Pelabuhan Tg.Priok	North Jakarta	12	25
	RS Islam Jakarta Utara	North Jakarta	15	
	RS Khusus Paru Firdaus	North Jakarta	5	
Cluster 3	RS Islam Jakarta Timur	East Jakarta	7	55
Cluster 4	RS Tugu Ibu	East Jakarta	6	20
	RSU Pasar Rebo	East Jakarta	31	
	RSU Haji Jakarta	East Jakarta	6	
Cluster 5	RS Puri Indah	South Jakarta	15	25
	RS Medika Permata Hijau	West Jakarta	3	
	Grha Kedoya	West Jakarta	12	

Information regarding bed allocations of each alternate hospital are available in Table 8. In cluster 1, two alternate hospitals were very far from being able to overcome the demand. Similarly, in cluster 3, RS Islam Jakarta Timur could not fulfil the required bed allocations. While for other clusters, at this period, the alternate hospital could provide the services needed for unassigned patients of COVID-19. However, this situation needs to be updated due to the dynamics of COVID-19 cases in Jakarta. When for developing new hospitals is not an option, then the Provincial Government of DKI Jakarta needs to allocate more additional health equipment in each alternate hospital. Otherwise, they need to search for other health care facilities nearby to be facilitated in providing services to COVID-19 patients.

### Standard deviational ellipse (SDE) modelling

**Fig. 12:**
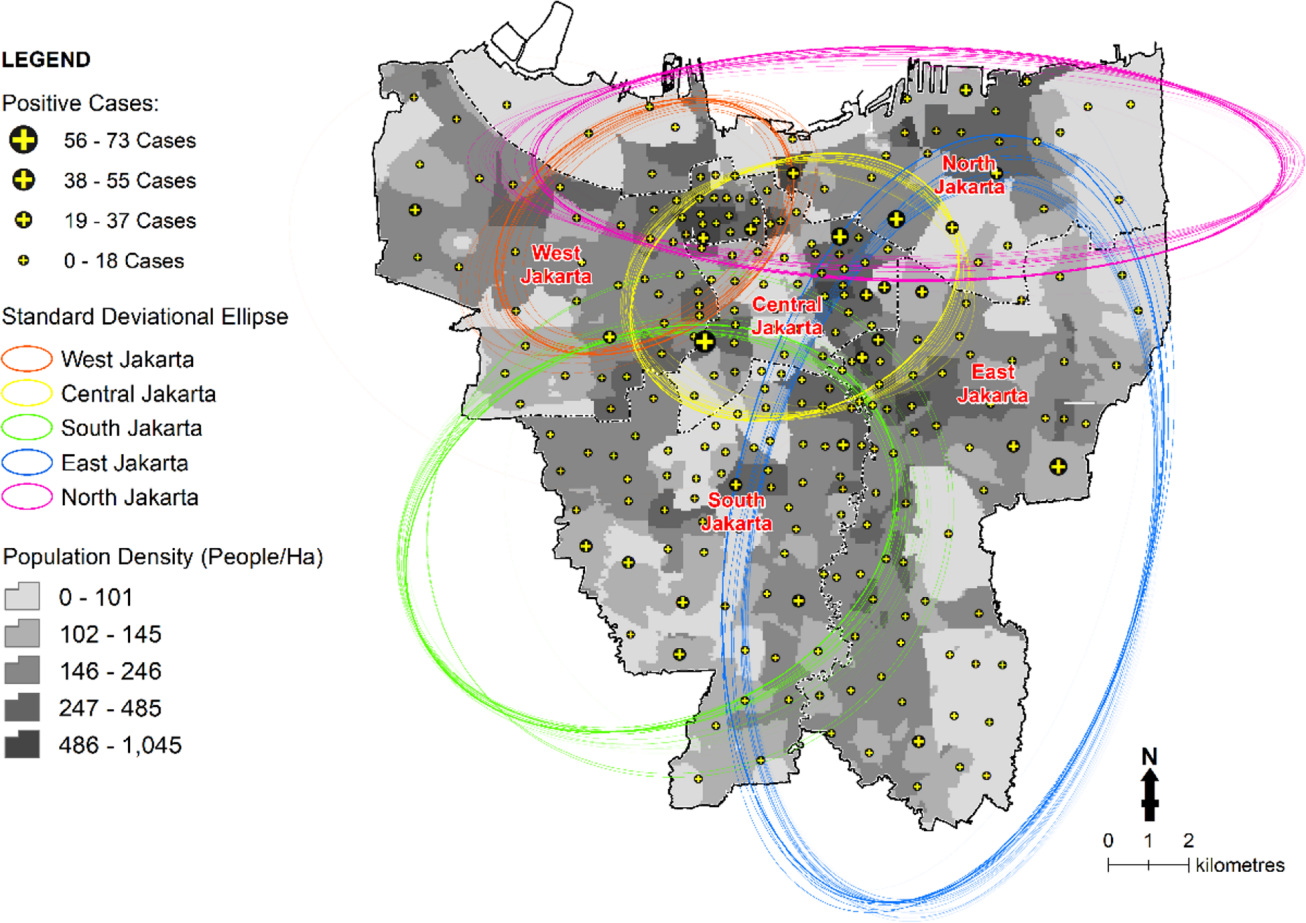
Standard Deviational Ellipse indicating trend of COVID-19 spread during March 25 – April 16, 2020 in five districts of DKI Jakarta Province. (source: own elaboration, 2020)

The shape of the study region as parent distribution is an important factor which may affect the shape and orientation of a point distribution and its ellipse. If the point set is a measure distributed over only part of the base, it is likely that the boundary shape has little effect. However, calculations of point distributions, which cover a whole sample area, must take the shape of that region into consideration [53]. In this study, we set the distribution area in the districts of DKI Jakarta Province. In accordance with several trials we have done, we specified two standard deviation (2 StDev) as an adjustment factor in order to generate ellipses containing 95% of the data points. The results of SDE model from confirmed number of the COVID-19 cases in each district in DKI Jakarta Province show various elliptical geometry values. These ellipses represent where was the most severe region of COVID-19 occurring. Spreading of the cases illustrates in ellipses encompassing the distribution of features and hence has a particular orientation. Their sizes and directions vary in each district (see Fig. 12) underlining the geographic distribution of COVID-19 cases in these areas. Apparently, most of the confirmed cases of COVID-19 occurred in Central Jakarta and the nearby area showing by the yellow ellipse as the centre of distribution since the district located in the most overlap area of others ellipses. Furthermore, these confirmed cases of COVID-19 appeared in densely populated area such as Kemayoran and Petamburan villages in Central Jakarta (360 people/ha), Matraman in East Jakarta (500 people/ha), Duri Kepa in West Jakarta (540 people/ha), and Pademangan Barat in North Jakarta (550 people/ha). This must be related to people movement since Jakarta has a dense population and has transportation links, including airplanes, trains, interstate buses, and private transportation modes.

**Fig. 13:**
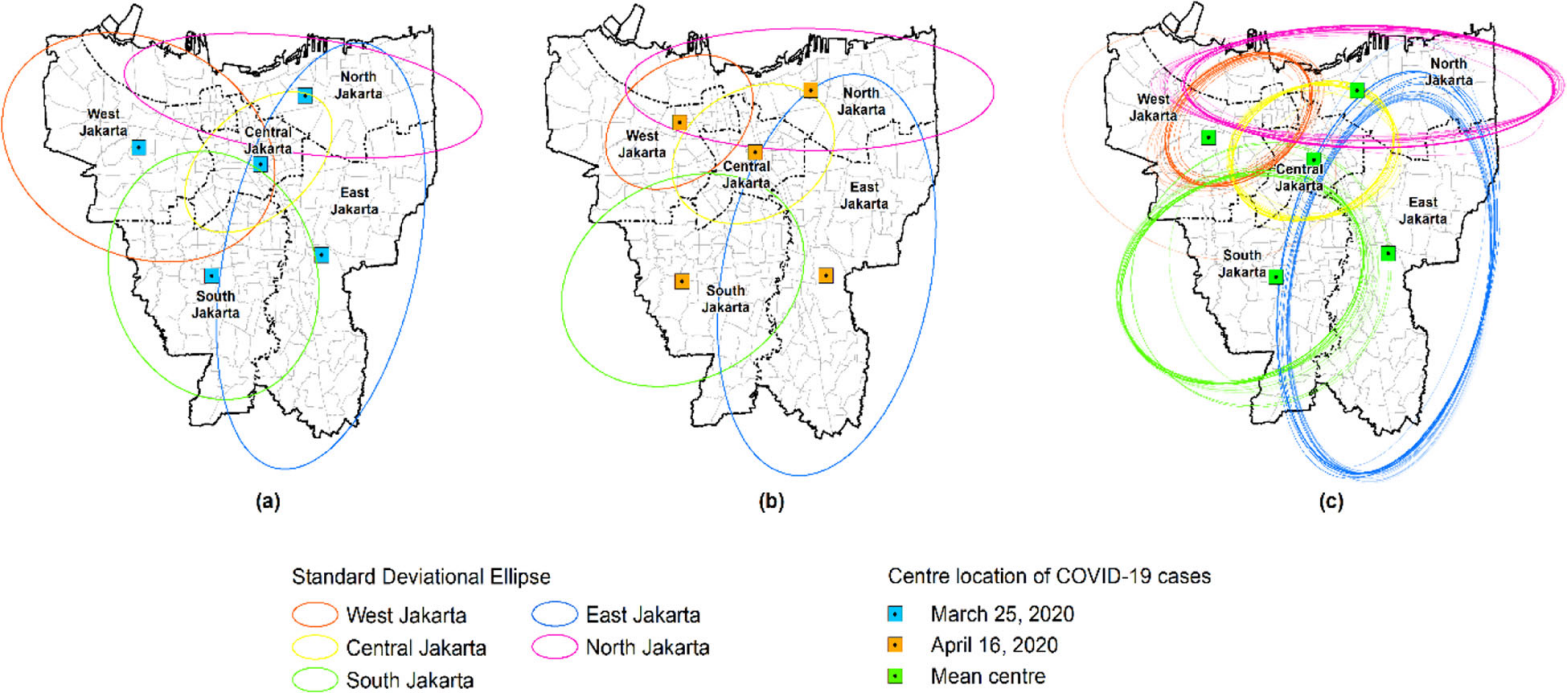
Centre locations of each Standard Deviational Ellipse of COVID-19 cases (**a**) March 25, 2020; (**b**) April 16, 2020; and (**c**) the mean centre based on all observed data in five districts of DKI Jakarta Province. (source: own elaboration, 2020)

We provided the characteristics of these standard derivational ellipses by area, and as a comparison, we presented two cases in March 25, and April 16, 2020. The centre locations of the two cases changed during the observed period (see Table 9). In West Jakarta, the centre location changed from Kedoya Utara to Jelambar Village; while in Central Jakarta, it moved from Kwitang to Senen village. Furthermore, in South Jakarta, the centre location shifted from Cipete Utara to Gandaria Selatan. In contrast, in the East and North Jakarta, the centre locations remained in the same villages, namely Halim Perdana Kusuma and Papanggo villages, respectively (see Fig. 13a and b). Similarly, the ellipse orientations were also changed from March 25, to April 16, 2020. A larger change was obvious in West Jakarta and South Jakarta (see Fig. 13a and b), while the ellipse direction of other areas was only slightly changed (see Table 9 and Fig. 13). A detailed visualisation of the change of ellipse directions is provided in Additional file 2. From the ellipse sizes, we can see that the ellipse size of West Jakarta became smaller in size. On the other hand, the ellipse size of South Jakarta was getting larger. These imply that the dispersion and spatial orientation of the deviational ellipses changed according to the trend of the number of confirmed cases periodically.

**Table 9:** The characteristics of standard deviational ellipses by area

Period	District	Centre Location		Epidemic Ellipse Size		Ellipse Orientation
		CentreX (m)	CentreY (m)	XStdDist (m)	YStdDist (m)	Rotation (° from North)
March 25	West Jakarta	694,226.737	9,317,384.245	11,385	8266	111.337
	Central Jakarta	703,932.120	9,316,248.077	6881	4135	51.542
	South Jakarta	700,261.416	9,307,612.422	8291	9664	157.463
	East Jakarta	708,951.968	9,309,076.182	7762	16,582	13.305
	North Jakarta	707,571.865	9,321,300.382	14,583	4412	97.452
April 16	West Jakarta	697,391.319	9,319,328.016	6460	4447	57.731
	Central Jakarta	703,354.907	9,316,901.414	6819	4975	66.294
	South Jakarta	697,626.224	9,307,303.675	10,167	7653	66.363
	East Jakarta	709,257.773	9,307,660.170	8406	15,514	11.979
	North Jakarta	707,933.690	9,321,782.895	14,898	4649	90.909

We summarize the average result of SDE model for the observed time series from March 25, to April 16, 2020 (see Fig. 13c and Table 10). The mean centres of the ellipse were finally located in Kedoya Utara village (West Jakarta), Kwitang (Central Jakarta), Cipete Utara (South Jakarta), Halim Perdana Kusuma (East Jakarta), and Papanggo (North Jakarta) as can be seen in Fig. 13c. In addition, the sizes and the rotations of each ellipse are provided in Table 10.

**Table 10:** The average results of the characteristic of standard deviational ellipse by each district

Period	District	Centre Location		Epidemic Ellipse Size		Ellipse Orientation
		CentreX (m)	CentreY (m)	XStdDist (m)	YStdDist (m)	Rotation (° from North)
March 25 to April 16, 2020	West Jakarta	694,951.55	9,318,046.89	11,842.15	7767.80	109.58
	Central Jakarta	703,503.65	9,316,290.16	7315.67	4111.07	63.42
	South Jakarta	700,391.06	9,307,373.39	9110.85	10,553.19	12.51
	East Jakarta	709,274.70	9,309,155.44	8266.66	16,606.24	11.97
	North Jakarta	706,884.40	9,321,604.29	16,026.11	5270.10	97.84

## Discussion

This study introduced geographic distribution of COVID-19 based on GIS spatial technology and recommend the alternate healthcare facilities by service area to support the existing referral hospitals. We compared two methods in deriving the service area, based on distance and travel time. The two methods show slightly different service area of referral hospitals. By using time-based service area, we obtained more realistic results and the estimation of time needed for patients to reach the nearby referral hospital. As mentioned by van Wee [54] to include the actual traffic conditions to the time component when generating service area is important, especially for COVID-19 patients who might experience the shortness of breath and severe fever. Moreover, in the case of emergency services, time it takes to receive a diagnosis is directly related to survival. Meanwhile, previous study has shown that the increasing distance from a service is related to the lower willingness of patients to come to that hospital. In the case of COVID-19 in Indonesia, some patients who have recovered, testified that they preferred to be hospitalized at the nearby hospitals rather than at suggested hospital which was far away from their homes, as also mentioned by Turnbull et al. [55] that people prefer to reach health-care facilities nearby their neighbourhood. Therefore, the government of DKI Jakarta Province needs to take these into consideration when selecting alternate hospitals for COVID-19 patients.

To our knowledge, efforts have been made by the Indonesian government to inform the public regarding the need to assign COVID-19 patients to referral hospitals and to ensure the capability of those referral hospitals in handling the COVID-19 patients. First was the implementation of the Decree of Minister of Health [15] on the definition of the health service criteria including the criteria for outpatients and the criteria for inpatients of COVID-19. Second was the implementation of the Decree of Minister of Health [14] on the guidelines for the prevention and control of COVID-19 on the national level. Third was planning and monitoring the availability of appropriate logistics to ensure the supply of medical devices including personal protective equipment, ventilators, medical indications (laboratory supplies) and medicines. In this case, the Ministry of Health monitors the chain of medicine distribution on the national scale, while the Provincial and District Health Offices not only ensure that the drug supply meets the needs of the primary and referral health facilities, but also are expected to anticipate delays in logistics delivery due to travel restrictions attributed to COVID-19 pandemic through an early preparation of drug requests as soon as possible and more routinely.

The OD cost matrices used least-cost paths yet obtained the information on the best routes from multiple origins to multiple destinations. The lines on Fig. 9 representing path and attribute tables have the ability to contain information about each line. But, since we have difficulties to manage the network road dataset from available data resources, in this map we did not perform analysis for cut off of time using OD Cost Metrix, since we cannot determine how many traffic lights, U-turn, and one-way or two-way road type along road network, furthermore, Jakarta have a policy to restricted odd and even number in certain day in a week (plate-car-number restriction). So, we used cut off of distance using OD Cost Metrix only. OD cost matrix solver output represented as the straight lines on our map, but the values stored in the lines attribute table reflect the network distance from origins to destinations. The matrix output is a table which lists the total impedance (could be a distance, time, or money) for the shortest path along the network between each centroid of COVID-19 patients (origins) and each referral hospitals as a demand points (destinations). The distance that is stored in the origin-destination cost matrix is calculated over the street network. Each demand point is assigned to the facility its closest to.

Search tolerance that we used to locate the input features on the network is about 5 m with rural driving distance mode with cut off from origin to destination is around 10 km. We chose rural driving distance because this mode defines the movement of cars and other similar small automobiles, such as pick-up trucks, and finds solutions that optimize travel distance. This travel obeys one-way roads, avoid illegal turns, and follows other rules that are specific to cars, but does not discourage travel on unpaved roads, with restrictions to avoid carpool roads, avoid express lane, avoid gates, avoid private roads, driving an automobile, roads under construction prohibited, through prohibited [33]. There is no set number of destinations to find. So, we can get the rank of distance from origin in a distance of 10 Kilometres to all possible destinations.

The SDE model was developed under assumption that the observed COVID-19 cases follow the normal distribution meaning that the cases are densest in the centre and becoming less dense toward the periphery. Hence, the use of SDE tool must considered the variance of the data and the shape of the study area as also have been mentioned by Wang et al., [27]. In this research, based on our experiment, we defined two standard deviation (2 StDev) as magnification factor can intensify confidence level until 95% of entire observed data i.e., COVID-19 cases. Failing in choosing this standard deviation parameter will result in errors since the SDE model may not represent the dispersion patterns and directions of COVID-19 cases in Jakarta. Therefore, SDE method must be used with a circumspection when calculating the geographic distribution of the features concerned. For instance, the delineation of a region concerned by SDE will not indicate the limits of the data spreading, but may yield unclear results from other features of the distribution.

Each of the deviational ellipse not only represented the direction and orientation of the COVID-19 outbreak during the time of observation, but also presented the compactness of the features. Furthermore, the area of those ellipses indicated data concentration. When the size was small relative to the study area, the point set was clustered, for instance, in Central Jakarta. Meanwhile, when the size was large, for instance, in East Jakarta, the data were widely distributed in the area.

## Conclusions

Based on the demand in contrast to the capacity of Referral Hospitals and unassigned positive-infected cases generated from OD Cost Matrix in Jakarta in a radius of 10 Kilometres from patients’ centroid, we can conclude if there is a need for developing referral hospitals or to allocate more additional health equipment in each alternate hospital. Otherwise, they need to search for other health care facilities nearby to be facilitated in providing services to COVID-19 patients that we have clustered by service area analysis. Unassigned positive-infected cases are highly concentrated in West Jakarta. West Jakarta has the highest population density in the amount of 19,516 people/km^2^. But apparently, based on the spatiotemporal concentration of COVID-19 cases that we have evaluated in 23-days based on data report, the concentration will grow toward Central Jakarta and the nearby area showing by the yellow ellipse as the centre of distribution since this region located in the ellipse of all regions. The SDE model can be used to investigate dispersion patterns and directions of COVID-19 cases and to identify risk factors during the certain period. Therefore, as disease control program, SDE model can be administered based on specific information in order to support effective health decision. Furthermore, COVID-19 patients and health workers must put efforts together to reduce COVID-19 transmission in crowded settings and in health care services according to the recommendations of the Indonesian Ministry of Health and WHO. Queueing in services must be minimized especially in places where patients tend to gather such as registration counters, laboratory queues and in the pharmacy to take their drugs.

There are several limitations in our study. First, the analysis in this study was limited to be based on the centroid of the villages which maybe less accurate than the patient’s home address. It might cause a potential to reduce the accuracy of the results, as it might influence the route taken affecting the distances and travel times. Second, with a small number of confirmed cases (from March 3, 2020, to April 16, 2020), our SDE model might not represents the real distribution patterns and directions of COVID-19 case. The limitations of the implementation of OD cost matrix in this research are: 1) the OD cost matrix generates results more quickly but cannot return the true shape of routes or their driving directions, 2) the OD cost matrix analysis solved least-cost paths along the network, it represents the origin-destination path as a straight line to visualize the matrix in the map, but the actual network paths cannot be displayed in the map, 3) the links to the actual on-line data cannot be provided, since the software that we used does not permit the reproduction of the results. But, its positivity on our research is the OD cost matrix solver reduces the computation time from each origin to all destinations and helps us by its destination rank to categorize and identify unassigned positive-infected cases and where it is located.

## Supplementary Information

**Additional file 1.** The capacity of the Referral Hospitals.

**Additional file 2.** Standard Deviational Ellipse per day starting from March 25, 2020 to April 16, 2020. Data and information regarding patients and COVID-19 trends in Jakarta can be directly accessed on https://corona.jakarta.go.id/id/data. For topographic map and hospital information, even the data is public domain, it requires Foreign Research Permit for international readers or users who want to use the data.

## Data Availability

Open access data direct to primary resources https://corona.jakarta.go.id/id/data. For topographic map and hospital information, even the data is public domain, it requires Foreign Research Permit for international readers or users who want to use the data.

## Abbreviations

OD Cost Matrix: Origin-Destination Cost Matrix
SDE: Standard Deviational Ellipse
GIS: Geographical Information System
PHEOC: Public Health Emergency Operating Center
ARI: Acute Respiratory Infection
PCR: Polymerase Chain Reaction
KITAS: *Kartu Izin Tinggal Terbatas* (Temporary Stay Permit Card in Indonesia)
NIK: Indonesian citizenship identification number
ICU: Intensive Care Unit
PICU: Paediatric intensive care unit
NICU: Neonatal Intensive Care Unit
ICCU: Intensive Coronary Care Unit
HCU: High Care Unit
