# Supplementary material for: GIS-based approaches on the accessibility of referral hospital using network analysis and the spatial distribution model of the spreading case of COVID-19 in Jakarta, Indonesia
Source: BMC Health Serv Res. 2020 Nov 20;20:1053. doi: 10.1186/s12913-020-05896-x (PMC7677106; doi:10.1186/s12913-020-05896-x)
Supplement: Supplementary file 1 — Additional file 1. The capacity of the Referral Hospitals. [file 12913_2020_5896_MOESM1_ESM.docx]

**Appendix 1.** The capacity of the Referral Hospitals

| ID | Referral Hospital | IR | ED | ICU | PICU | NICU | ICCU | HCU | LUNG | RADL | THO | RADN | RADG | NERS |
| --- | --- | --- | --- | --- | --- | --- | --- | --- | --- | --- | --- | --- | --- | --- |
| 60 | RSPI Sulianti Saroso | 14 | 12 | 3 | 0 | 0 | 0 | 5 | 6 | 2 | 0 | 9 | 0 | 2 |
| 61 | RSUP Persahabatan | 18 | 10 | 13 | 0 | 2 | 5 | 4 | 25 | 8 | 4 | 10 | 5 | 8 |
| 62 | RSUP Fatmawati | 54 | 40 | 17 | 3 | 4 | 17 | 40 | 5 | 6 | 1 | 11 | 0 | 10 |
| 63 | RSPAD Gatot Soebroto | 6 | 22 | 12 | 2 | 4 | 0 | 27 | 9 | 16 | 0 | 0 | 0 | 0 |
| 64 | RSUD Cengkareng | 30 | 15 | 5 | 0 | 3 | 4 | 20 | 3 | 4 | 1 | 1 | 0 | 3 |
| 65 | RSUD Pasar Minggu | No Data | | | | | | | | | | | | |
| 66 | RSU Bhayangkara Tk. I R.Said Sukanto | 17 | 32 | 44 | 0 | 9 | 5 | 0 | 3 | 3 | 1 | 0 | 0 | 0 |
| 67 | RSAL Mintoharjo | 0 | 12 | 7 | 0 | 0 | 5 | 0 | 3 | 5 | 0 | 0 | 0 | 0 |

Source: Indonesian Ministry of Health, 2020

Note:

IR= Isolation room; ED=Emergency Department; ICU= Intensive Care Unit; PICU= Paediatric intensive care unit; NICU= Neonatal Intensive Care Unit; ICCU= Intensive Coronary Care Unit; HCU= High Care Unit; LUNG= Lung specialist; RADL= Radiologist specialist; THO= Thorax specialist; RADN= Radiation therapist; RADG= Radiographer; and NERS= National certified nurse.
