# Supplementary figures and images for: GIS-based approaches on the accessibility of referral hospital using network analysis and the spatial distribution model of the spreading case of COVID-19 in Jakarta, Indonesia
Source: BMC Health Serv Res. 2020 Nov 20;20:1053. doi: 10.1186/s12913-020-05896-x (PMC7677106; doi:10.1186/s12913-020-05896-x)

**Appendix 2.** Standard Deviational Ellipse


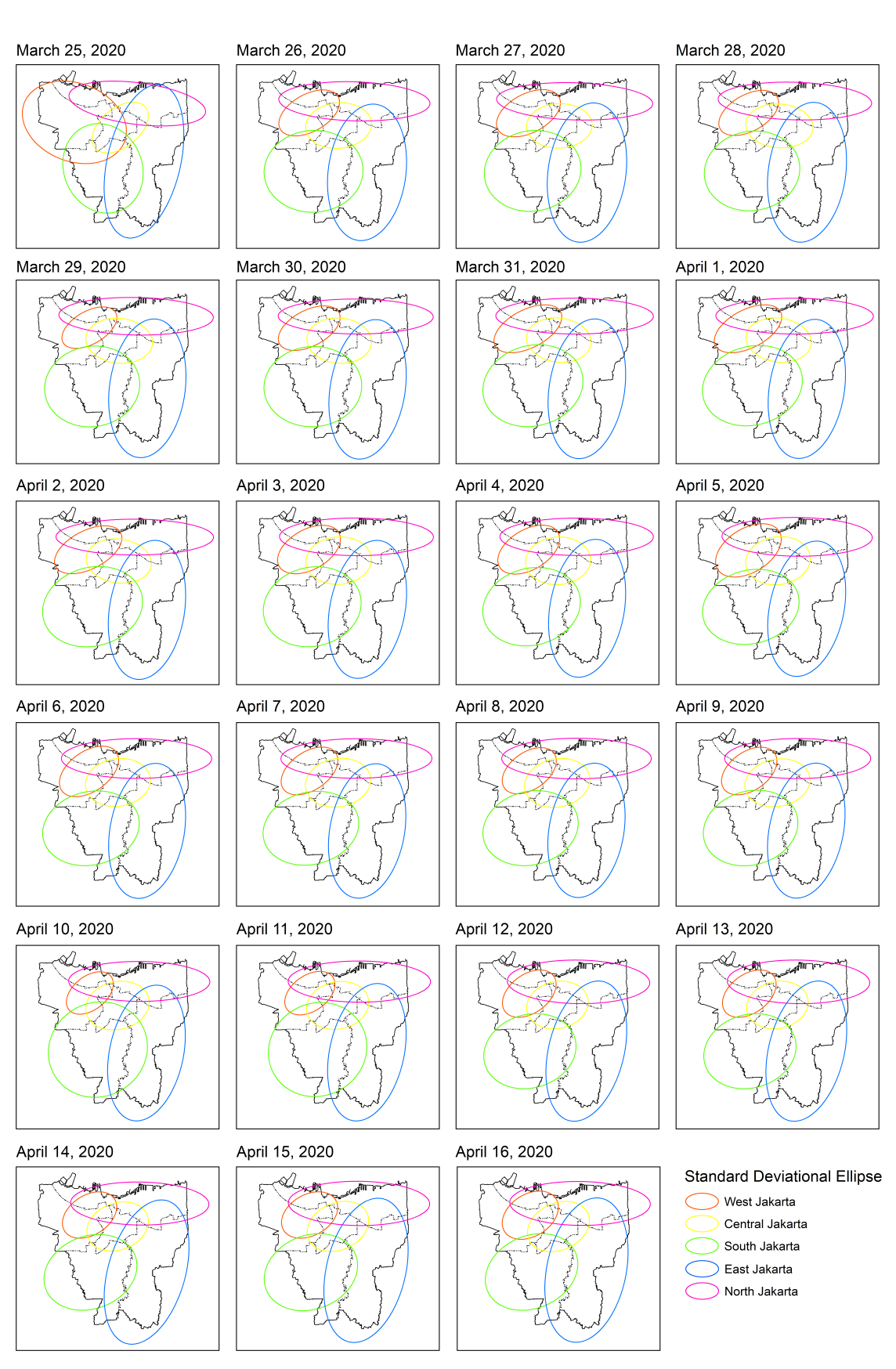

Supplement: Supplementary file 2 — Additional file 2. Standard Deviational Ellipse per day starting from March 25, 2020 to April 16, 2020. Data and information regarding patients and COVID-19 trends in Jakarta can be directly accessed on https://corona.jakarta.go.id/id/data. For topographic map and hospital information, even the data is public domain, it requires Foreign Research Permit for international readers or users who want to use the data. [file 12913_2020_5896_MOESM2_ESM.docx]
